# Phenolic contents, antimicrobial and antioxidant activity of *Olea ferruginea* Royle (Oleaceae)

**DOI:** 10.1186/s12906-018-2239-0

**Published:** 2018-06-05

**Authors:** Ansar Mehmood, Ghulam Murtaza

**Affiliations:** 1 Department of Botany, University of Poonch, Rawalakot, Azad Jammu and Kashmir 12350 Pakistan; 2 Department of Botany, University of Azad Jammu and Kashmir, Muzaffarabad, 13100 Pakistan

**Keywords:** Extract, *E. coli*, Ethnopharmacology

## Abstract

**Background:**

*Olea ferruginea* Royle (Oleaceae) has long been used as an important ethnomedicinal plant to cure fever and debility, toothache, hoarseness, throatache and skeleton disorders. In this study, phenolic contents, antimicrobial and antioxidant activities of leaf and bark extracts (chloroform, ethanol and methanol) of *O. ferruginea* were evaluated.

**Methods:**

Total phenolic contents were determined by Folin-Ciocalteu Spectrophotometric method*.* Antimicrobial activity was examined against *Bacillus subtilis* and *Staphylococcus aureus* (Gram positive)*, Escherichia coli* (Gram negative), *Candida albicans* and *Sccharomyces cerevisiae* (yeas strains) by disc diffusion method. Antioxidant activity was observed through DPPH assay*.*

**Results:**

The higher phenolic content was found in bark extract (376 μg/mg) of *O. ferruginea*. Chloroform extracts was found inactive against tested microorganisms while ethanol and methanol extracts showed pronounced inhibitory activity against both gram positive and gram negative bacteria. Only methanol extract of leaves inhibited the yeast strains. None of the bark extract inhibited the growth of tested yeast strains. The zones of inhibition formed by plant extracts were compared with zones of inhibition of available reference antibiotic discs such as tetracycline, ciprofloxacin and nystatin. Higher antioxidant activity was observed with methanol extracts of leaves and bark of *O. ferruginea*.

**Conclusion:**

These findings show that *O. ferruginea* has potential antimicrobial and antioxidant activities. This study suggests a possible application of olive leaves and bark as sources of natural antimicrobial and antioxidants.

## Background

Medicinal plants constitute a large group of economically significant plants having raw materials for the synthesis of medicines, flavors, perfumes and cosmetics. The products of these plants serve as valuable foundation of income for small owners and also add valuable foreign exchange for a country by export. No doubt, anti-microbial agents are the most significant therapeutic findings of the twentiethcentury. However, with the extensive use of antibiotics, human is now facing a problem of developing resistance in almost all pathogens [1].

Various type of sources such as microorganisms, plant, animals and oils have been explored to discover the new antimicrobial agents. The systematic screening of such sources like folk medicine result in the finding of innovative effective compounds [2]. Folk medicines are great source not only for curing health of the poor in developing countries but also in developed countries where conventional medicines are predominant for national health care [3]. The pathogens developed resistance to antibiotics which opened the door to use herbal medicines as antimicrobial agents [4].

Plant synthesized and produced different types of secondary metabolites which possess antimicrobial activity [5]. Formerly it was thought that secondary metabolites, not the products of the primary metabolic pathway, have no advantage to the plants who produced them. However, now it is believed that they containvigorous functions [6]. The search for new antibiotics which can replace conventional antibiotics is a need of hour. To meet this need present work was carried out to scan the antimicrobial and antioxidant activity of a valuable medicinal plant such as *O. ferruginea.*

Olive tree (*O. ferruginea* Royle), covers 8 million hectares in Mediterranean countries almost 98% of the world crop, is one of the most important fruit trees [7]. These figures show its pronounced economic and social meaning and the probable aids to be derived from exploitation of any of its byproducts [8]. The fruits and oil of *Olea*, important constituents of daily diet in a large part of the world’s population, are widely studied for their alimentary use, whereas the leaves contain important secondary metabolites like oleuropein and oleacein, the former responsible for hypoglycemic activity [9] and the latter for hypotensive activity [10]. It was also shown by several studies that leaf extract of olive has the ability to reduce the blood pressure in animals [11], prevent intestinal muscle spasms and relieve arrhythmia [12]. Present work was aimed to investigate the phenolic contents, antimicrobial and antioxidant activity of *O. ferruginea*.

## Methods

### Plant materials

In this study, *Olea ferruginea* (stem bark and leaves) was selected for its antimicrobial and antioxidant potential. The plant was collected from Kotli in 2017 and identified by a taxonomist Dr. Sajjad Hussain, Department of Botany, University of Poonch Rawalakot. The voucher specimen (KNV 416) was submitted in the Herbarium, University of Poonch Rawalakot. The plant material was obtained in the course of flowering stage. Both bark and leaves were shade dried at room temperature (25 ± 2 °C).

### Extraction procedure

After drying, a fine powder of bark and leaves was made using electric grinder. For extraction, Fifty g powder was soaked with 200 ml of chloroform, ethanol and methanol solvents in three separate flasks. The maceration was carried out at room temperature in each solvent for 7 days with constantly shaking after every 24 h. After maceration, the mixture was filtered using Whatmann filter paper in labeled flasks. The filtrate was evaporated at low temperature and pressure by a rotary evaporator to obtain the crude extract [13].

### Dilution

Ten mg crude extract was dissolve in 1 ml respective solvents (chloroform, ethanol and methanol) to make 10 mg/ml dilution.

### Microorganisms

All the tested bacteria (*Bacillus subtilis*, *Staphylococcus arureus* and *Escherichia coli*) were obtained from Combined Military Hospital (CMH) Muzaffarabad while the tested yeasts (*Candida albicans* and *Sccharomyces cerevisiae*) were grown in Laboratory, Department of Botany, University of Azad Jammu and Kashmir, Muzaffarabad.

### Culture media

Nutrient agar medium (28 g dehydrated nutrient agar in 1000 ml distilled water, warmed and shake) was used for culturing bacterial species. The fungal species were cultured in Sabouraud’s dextrose agar (65 g Sabouraud’s dextrose agar in 1000 ml distilled water). Both the media were autoclaved for 15 min at 121 °C.

### Antimicrobial assay

Disc diffusion essay proposed by [14] was used to test the extracts of plants for their antimicrobial activities. The microorganisms were suspended in 10 ml distilled water by dipping a loop of organism in sterilized labeled test tube. From test tube, 1 ml dilution was transported in the corresponding sterilized petri plates. The dilution and medium were mix in petri plates by gently shaking and kept at room temperature for solidification.

Sterilized filter paper discs of 6 mm in diameter were dipped in each 10 mg/ml crude extracts (chloroform, ethanol and methanol) of *O. ferruginea* and placed on agar medium in petri plates at their labeled positions. Commercially available antibiotics (Tetracyline, ciprolaxacine, and nystatin) were used as positive control and water, chloroform, ethanol and methanol as negative control. The experiment was performed in aseptic environment.

### Incubation of plates

The plates containing the bacterial and yeasts culture were incubated at 37 °C for 24 h and 25 °C for 72 h respectively. The zones of inhibition were measured in millimeter by using measuring scale.

### Antioxidant activity

To determine antioxidant activity of selected plants, the DPPH (2,2-diphenyl-1-picrylhydrazyl) radical scavenging assay [15] was used to determine the antioxidant activity of different extracts of leaves and bark of *O. ferruginea*. The DPPH solution fades its color when received hydrogen ions from antioxidant, which was initially violet. A stock of DPPH was prepared by adding 7 mg DPPH in 100 ml of methanol. The DPPH solution, methanol and extracts with various concentrations (1.25 mg/ul, 2.5 mg/ul and 5 mg/ul) were added in labeled test tubes for sample and blank reading, mixed well and kept it for 30 min at room temperature. Ascorbic acid was used as control. The optical density was measured against standard at λ_max_ 517 nm by using UV visible spectrophotometer. The experiments were carried out in triplicate. The percentage radicals scavenging activity was calculated by using following formula

$$ \%\mathrm{Inhibition}=\left[\frac{\mathrm{Absorbance}\ \mathrm{of}\ \mathrm{control}-\mathrm{Absorbance}\ \mathrm{of}\ \mathrm{tested}\ \mathrm{sample}}{\mathrm{Absorbance}\ \mathrm{of}\ \mathrm{control}}\right]\times 100 $$

Where standard is the absorbance of control reaction (containing all reagents except the test compounds). 50% inhibition (IC50) of each extract concentrations against graph of inhibition was calculated by applying SSP10 software.

### Phenolic estimation

Folin-Ciocalteu Spectrophotometric method described by Kim et al. [16] was used to determine the total phenolc content of plant extracts on a UV-vis spectrophotometer at 650 nm. Results were expressed as catechol equivalents (μg/mg).

### Statistical analysis

A statistical analysis was used to interpret the antimicrobial and antioxidant results. The experiment was conducted in completely randomized design with 3 replicates. The results are presented as means ±standard error of means using MS excel [17].

## Results

### Antimicrobial activity

**Fig. 1:**
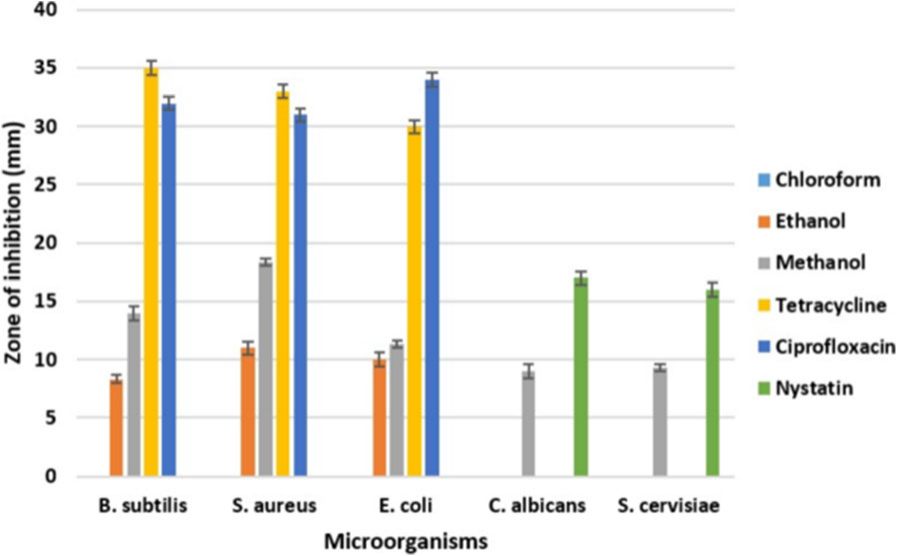
Antimicrobial activity of crude extracts of *O. ferruginea* (leaves) compared to antibiotics

**Fig. 2:**
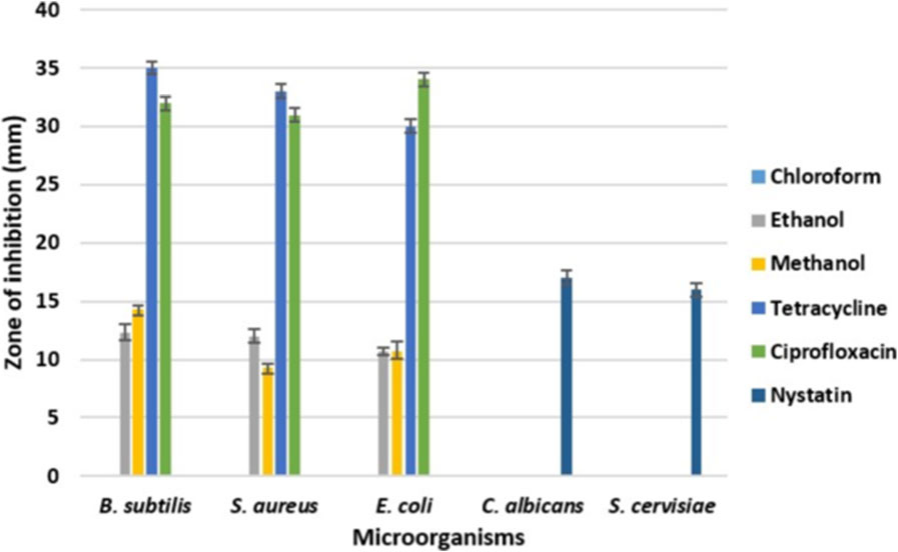
Antimicrobial activity of crude extracts of *O. ferruginea* (stem bark) compared to antibiotics

**Fig. 3:**
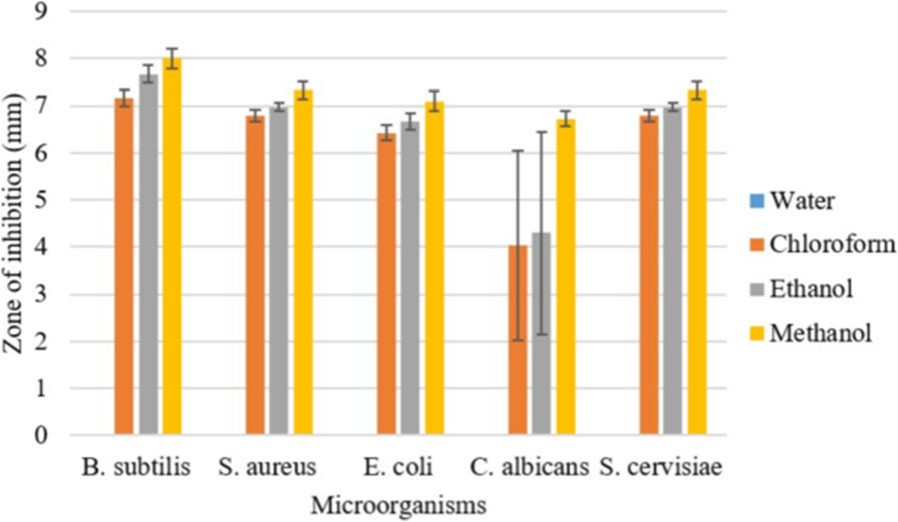
Antimicrobial activity of negative controls

Figure 1 shows the antimicrobial activity of crude leaf extracts of *O. ferruginea* while Fig. 2 shows the antimicrobial activity of crude bark extracts of *O. ferrugnea*. The antimicrobial activity of negative controls such as water, chloroform, ethanol and methanol is shown in Fig. 3. The chloroform extract from both leaves and bark did not inhibit the growth of any of the tested organisms and found to be inactive. Ethanol leaf extract was less active against *B. subtilis* i.e. 8.33 ± 0.33 mm while it showed appreciable bactericidal activity against *S. aureus* and *E. coli* i.e. 11.00 ± 0.58 mm and 10.00 ± 0.58 mm. While *C. albicans* and *S. cerevisiae* were resistant to ethanolic leaf extract. Ethanol bark extract showed good inhibitory activity against *B. subtilis* (12.33 ± 0.67 mm) and *S. aureus* (12.00 ± 0.58 mm) while it was moderately active against *E. coli* (10.67 ± 0.33 mm). It was also unable to inhibit the growth of yeast strains.

Methanolic leaf extract induced higher antimicrobial activity against *B. subtilis* and *S. aureus* (14.00 ± 0.58 mm, 18.33 ± 0.58 mm) respectively. It also showed a considerable amount of activity against *E. coli* with zone of inhibition of 11.33 ± 0.33 mm. Methanol leaf extract also inhibited the growth of *C. albicans* and *S. cerevisiae* with zones of 9.00 ± 0.58 mm and 9.33 ± 0.33 mm. It was also observed that Gram positive bacteria are more susceptibleto the tested extracts than gram negative bacterium. The highest activity (18.33 ± 0.58 mm) was found of methanol extract of leaves of *O. europaea* against *S. aureus*.

### Phenolic contents

Tables 1 and 2 shows the total phenolic contents in crude extracts of *O. ferruginea* and were reported as catechol equivalents (μg/mg). The higher phenolic compounds (376 μg/mg) were present in the methanol extract, followed by ethanol (321 μg/mg) and chloroform extract (288 μg/mg) of leaves of *O. ferruginea*. In stem bark extract, highest phenol was reported in methanol (399 μg/mg), followed by ethanol (351 μg/mg) and chloroform (312 μg/mg).

**Table 1:** Phenolic content and IC50 value in *O. ferruginea*leaves

Solvent used	Stem bark	
	Phenolic contents (μg/mg)	IC _50_ value (mg/ml)
Chloroform	312	0.84
Ethanol	351	0.56
Methanol	399	0.46

**Table 2:** Phenolic content and IC_50_ value in *O. ferruginea* stem bark

Solvent used	Leaves	
	Phenolic contents (μg/mg)	IC 50Value (μg/ml)
Chloroform	288	0.67
Ethanol	321	0.47
Methanol	376	0.37

### Free radical scavenging activity

**Fig. 4:**
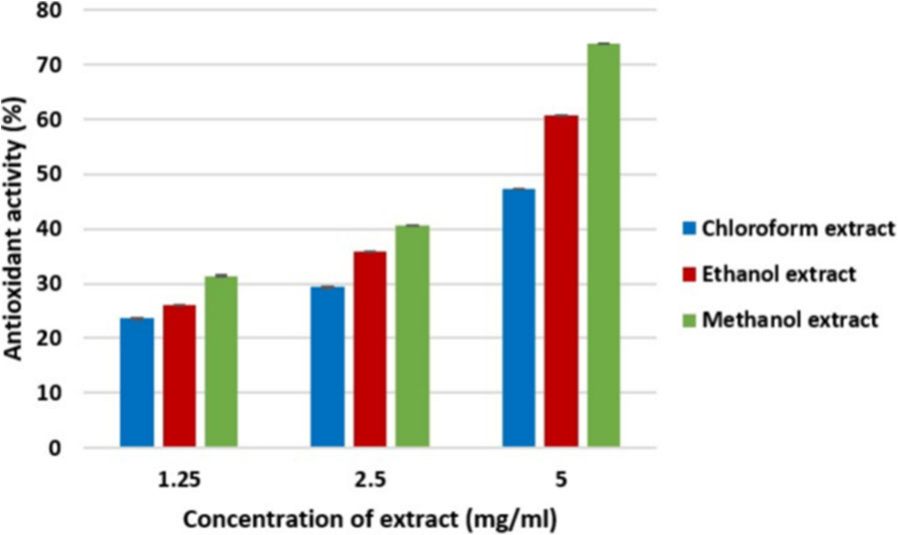
Antioxidant activity of *O. ferruginea* leaves in different solvents

**Fig. 5:**
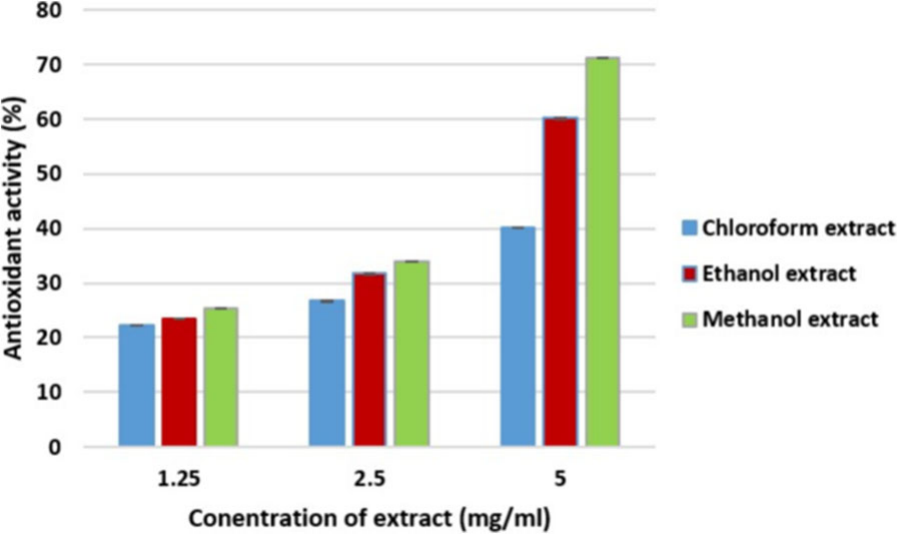
Antioxidant activity of *O. ferruginea* stem bark in different solvents

Free radical (DPPH) scavenging activity of leaves of *O. ferruginea* is shown in Fig. 4 in the form of percentage inhibition. According to Fig. 4, chloroform, ethanol and methanol showed 22.22 ± 0.03, 42.48 ± 0.05 and 25.26 ± 0.01 free scavenging activity respectively at 1.25 mg/ml. the extracts of 2.50 mg/ml concentration of chloroform, ethanol and methanol revealed 26.68 ± 0.02, 34.03 ± 0.03 and 49.71 ± 0.03 respectively. While chloroform, ethanol and methanol extracts at 5.00 mg/ml concentration showed 40.22 ± 0.06, 60.15 ± 0.03 and 71.24 ± 0.02 activity respectively. Figure 5 shows the results of the free radical (DPPH) scavenging activity in % inhibition of stem bark of *O. ferruginea*. The result suggested that the chloroform, ethanol and methanol extract of leaves exhibited antioxidant activities of 23.56 ± 0.01, 26.05 ± 0.02 and 31.41 ± 0.05 respectively at a concentration of 1.25 mg/ml. At concentration of 2.50 mg/ml, the chloroform, ethanol and methanol extract of leaves showed 29.34 ± 0.06, 35.82 ± 0.06 and 40.67 ± 0.03 antioxidant activity respectively. Similarly chloroform, ethanol and methanol extract at 5.00 mg/ml concentration showed antioxidant activity of 47.33 ± 0.06, 60.72 ± 0.03 and 73.97 ± 0.02 respectively.

### Half maximum inhibitory concentration (IC_50_ value)

Tables 1 and 2 also shows the IC_50_ value of chloroform extract (0.8 mg/ml), ethanol (0.55 mg/ml) and methanol extract (0.45 mg/ml) of leaves of *O. ferruginea*. While stem bark showed IC_50_ value of 0.67, 0.46 and 0.37 mg/ml with chloroform, ethanol and methanol extracts respectively.

## Discussion

Medicinal plants are the active source of both traditional as well as modern medicines. The active compounds present in herbal medicines have the compensations of being joined with several other compounds that seem to be inactive. However as compared to isolated and pure active compounds, the bio compounds present in plants give them far superior security and efficiency as a whole [18]. Many studies have been carried out to investigate the antibacterial and antifungal activities of essential oil of olive. The presented work was conducted to observe the antimicrobial activity of medicinal plant as *O. ferruginea* against gram positive bacteria, gram negative bacterium and yeast strains. From the results it was observed that Gram positive bacteria (*B. subtilis and S. aureus*) were more sensitive as compared to gram negative bacterium (*E. coli*) used. The results were well correlated to findings of [19] where gram negative bacterium *E. coli* was also more resistant to the extracts of *S. xanthocarpum*. It was also observed that methanol extract showed highest inhibitory activity because of its high polarity and it allows extracting all the phenolic compounds. In addition, as compared to isolated compounds, extracts can be more beneficial. Since in presence of other compounds in the extracts, an individual bioactive component can change its properties [20]. It was also found that n-hexane fraction of leaf of *O. ferruginea* has higher biological activity against gram positive and gram negative bacteria as compared to chloroform and methanol. While in our study methanol extract was found to have higher antimicrobial activity [21].

It is observed from present results that active antimicrobial biocompounds could be extracted in ethanol and methanol extracts. Except phenolics, bound to insoluble carbohydrates or proteins, most of the compounds extract in methanol or acetone [22]. In present study, ethanolic and methanolic extractions of *O. ferruginea* were found to have acceptable antimicrobial activities with respect to reference antibiotic discs (Fig. 1). It was observed in present study that plant extracts showed high inhibitory activity against bacteria in contrast to fungi. Previously extracts of *Bellis perennis* have also shown more activity towards bacteria [23]. Moreover, methanolic extract was found more active in our studies which is correlated to results of [24], methanol extract of *H. afficinalis* was more active against *P. aeruginosa* and *B. subtillus.* The antimicrobial activity of plants may be the consequence of presence of wide range of bioactive compounds. Plant extracts comprise many polyphenols, flavonoids and alkaloids which could be antimicrobial representatives. Many studies show the association between antimicrobial activities of plants and the phytochemicals present in them. Flavonoids are well known for their antiviral [25], antimicrobial [26] and spasmolytic [27] activities. Likewise, alkaloids extracted from plants have also shown antimicrobial activity [28]. The inhibition of microbial growth may result from the binding of biocompounds to the cell wall.

The antioxidant activity of plants and their natural products is widely evaluated by using scavenging activity for free radicals of 1.1-diphenyl-2-picrylhydrazyl (DPPH).The methanol extract of stem bark of *O. ferruginea* showed higher radical scavenging activity (73.97 ± 0.02) followed by ethanol and chloroform extracts. Overall, methanol extracts have shown higher antioxidant activity. It has already been proven that phenolic compounds are best extracted in ethanol and methanol solvents [29]. Total phenolic content was found higher in methanol extract as compared to ethanol and chloroform extracts. Therefore, it can be easily assume that DPPH free radical scavenging activity is corresponded to present of bioactive compounds like phenols, as methanol extract showed highest antioxidant activity. A significant variation was found in total phenolic content among the different extracts of stem bark and leaf extract of *O. ferruginea*. Free radicals such as superoxide, hydroxyl, peroxyl and singly oxygen play a vital role in many disease conditions. Herbal drugs, comprising free radical scavengers, are getting prominence in treating such diseases. Many plants shows competent antioxidant properties due to presence of phytochemicals including phenolic compounds [30].

The amount or concentration of substrate causes 50% loss of the DPPH activity is known as its IC50 and it is calculated by plotting a linear regression of antiradical activity in percentage against the amount of compounds tested. Methanol extracts showed being the lowest IC50 values which shows highest antioxidant activity. As compared to leaves extracts, stem bark extracts of *O. ferruginea* exhibited significant activity with low IC50 value. Moreover, a linear relationship was found between the total polyphenol and the reciprocal of IC50 value, indicating that polyphenols are directly proportional to antioxidant activity. Similar results were recorded by [31].

## Conclusion

*O. ferruginea* is known for treating many important infectious diseases including to kill cancerous cells. Different crude extracts of tested plants were prepared by using various solvents with the aim of screening better antimicrobial activities in comparison with some standard antibiotics. Among these, the extract obtained with methanol was found to have a better effectiveness against the tested bacteria and also has highest antioxidant activities. The results of present study support the use of this valuable plant in traditional medicines for treating infectious diseases. Further phytochemicals studies are needed to find the major bioactive compounds responsible for antimicrobial and antioxidant effect of this plant.
